# Advances in Drug Discovery for Cardiomyocyte Proliferation

**DOI:** 10.1007/s11936-025-01107-0

**Published:** 2025-07-19

**Authors:** Kaitlyn L. Wintruba, Matthew J. Wolf, Jop H. van Berlo, Jeffrey J. Saucerman

**Affiliations:** 1 Department of Biomedical Engineering, University of Virginia, Charlottesville, VA 22908 USA; 2 Division of Cardiovascular Medicine, University of Virginia, Charlottesville, VA 22908 USA; 3 Lillehei Heart Institute, Department of Medicine, University of Minnesota, Minneapolis, MN 55455 USA

**Keywords:** Cardiovascular Disease, Congenital Heart Disease, Cardiac regeneration, Cardiomyocyte proliferation, Drug discovery, Drug screens

## Abstract

**Purpose of Review:**

This review explores current advancements in drug discovery for promoting cardiomyocyte proliferation and highlights key challenges in translating these findings to clinical applications.

**Recent Findings:**

High-throughput screening platforms, including phenotypic assays using stem cell-derived or neonatal cardiomyocytes, have identified candidate compounds that modulate proliferative signaling pathways. Computational modeling and omics analysis have enabled mechanistic insights and supported the development of targeted drug discovery strategies. Emerging approaches are increasingly incorporating orthogonal screening and cross-species validation to improve translational potential.

**Summary:**

While no therapy has yet fully translated beyond pre-clinical models, significant progress has been made in identifying candidate drugs that stimulate cardiomyocyte proliferation in animal models. Translating these findings into effective therapies requires a rigorous foundation in basic research to clarify the molecular mechanisms of cardiac repair and guide drug development.

## Opinion Statement

Drug discovery for cardiac regeneration is a major challenge that can only progress based on a rigorous foundation of advancements in our understanding of cardiomyocyte proliferation. Cardiac regeneration is inherently complex, involving multiple cell types and varying across developmental stages and disease states. Discovering drugs that promote cardiomyocyte proliferation represents a promising strategy to treat adult and congenital diseases associated with defective cardiac regeneration.

One of the primary challenges in drug discovery is the difficulty in translating candidate compounds into effective therapies. Targeting a single molecular pathway often fails to elicit functional regeneration, as a complex interplay of signaling networks governs cardiac repair. Additionally, promoting cardiomyocyte proliferation presents inherent risks, including the potential for uncontrolled cell growth or off-target effects. Further complicating the process, in vitro models lack the structural and cellular complexity of native cardiac tissue, while animal models do not fully recapitulate human physiology, limiting both reproducibility and translational relevance.

Given the limitations of experimental platforms, no single approach is sufficient. Instead, a diverse and complementary set of approaches is essential to advancing the field. Orthogonal screening strategies offer a promising approach to overcoming challenges associated with cardiac regeneration drug discovery by enhancing validation and improving therapeutic outcomes. Computational and systems biology approaches leverage large-scale omics datasets, integrating predictive modeling to identify multi-targeting strategies that address the complexity of cardiac repair. Strengthening drug discovery pipelines by integrating diverse methods can reduce off-target effects, improve candidate selection, and ultimately accelerate the translation of basic research findings into clinical applications.

## Introduction

Cardiovascular disease remains the leading cause of mortality worldwide, with over 19 million deaths reported annually [1]. The irreversible loss of cardiomyocytes (CMs) that occurs during myocardial infarction (MI), and subsequent replacement by collagen and extracellular matrix in the infarcted myocardium leads to cardiomyopathy. A major factor contributing to the untoward sequelae of MI is the limited regenerative capacity of human CMs, with an annual CM renewal rate of less than 1%, as demonstrated by carbon-14 integration studies [2] and isotope labeling [3]. In contrast, CM proliferation drives heart growth and maturation during development [4]. The neonatal mouse heart exhibits a transient regenerative potential [5]; however, CM proliferation declines postnatally due to permanent cell cycle exit, metabolic shifts, and epigenetic modifications [6]. Importantly, dysregulated CM proliferation is implicated in the pathology of congenital heart defects (CHDs) [7], emphasizing its critical role in cardiac development. Developmental changes in CM structure and function, driven by age-dependent alterations in gene expression and signaling pathways, precede anatomical and functional remodeling of the heart, highlighting the need to understand proliferation dynamics for therapeutic advancements.

Current cardiac therapies do not directly target CM proliferation or regeneration. The standard of care for MIs, cardiomyopathies and heart failure includes β-blockers, ACE inhibitors, mineralocorticoid report antagonists, sodium-glucose transport protein 2 inhibitors, and surgical interventions, such as heart transplants. These therapeutics primarily manage symptoms and prevent disease progression rather than restoring lost cardiac tissue. The adult heart lacks endogenous stem cells, and exogenous stem cells often fail to engraft or regenerate functional myocardium. Furthermore, the absence of endogenous CM progenitors, such as c-Kit + cells, suggests that de novo CM formation through progenitor activation is unlikely [8]. While the delivery of CMs derived from stem cells offers potential [9], there are challenges associated with the electrical and mechanical integration into surrounding myocardium. Strategies to induce endogenous CM proliferation and intrinsic cardiac repair represent an attractive alternative. Developing drugs that modulate CM proliferation could offer therapeutic benefits for conditions such as CHD or MI, yet identifying effective, safe, and clinically translatable compounds remains a significant challenge. Preventing excessive proliferation of non-CM cell types or compromising myocardial contractility are major concerns that need to be addressed to develop viable therapies. In this review, we discuss recent advancements in drug screening for CM proliferation and regeneration, highlighting both the successes and the limitations of current drug discovery approaches.

## Methods and Successes in Drug Screening

Screening approaches enable the discovery of new drugs for cardiac regeneration by identifying candidates that promote CM proliferation while ensuring therapeutic efficacy, safety, and scalability. High-throughput screening platforms commonly utilize induced pluripotent stem cell-derived cardiomyocytes (iPSC-CMs) or neonatal CMs, typically measuring proliferation through one or two cell cycle markers (Table 1). Drug discovery strategies are not limited to traditional methods but also include innovative hybrid approaches that leverage both de novo development and repurposing of existing drugs.

**Table 1:** Summary of screens for cardiomyocyte proliferation

Type of screen		Screen measurement	# Hits	Drug or gene identified	ref.
small molecule	structure-based drug repurposing	MEIS1 and HOXB13 binding	107/8,600	paromomycin + neomycin (aminoglycosides)	[10]
	human PSC-cardiac organoids	Ki67, contractility	1/4	nifedipine (LTCC inhibitor)	[11]
			3/105	p38 inhibitor, TGFBR inhibitor	[12]
	human iPSC-CMs	nuclei count	20/429	palomid-529 (mTORC inhibitor)	[13]
			4/94	AS1842856 (FOXO inhibitor)	[14]
		EdU	68/5,094	nitrendipine, verapamil (LTCC inhibitors)	[15]
	neonatal rat CMs		3/64	TT10 (YAP agonist)	[16]
		FUCCI: geminin	13/11,000	ADRA1s agonist + JAK1, DYRKs, PTEN, and MCT1 inhibitors	[17]
			8/128	carbacyclin (PPARδ agonist)	[18]
	mouse ESCMs		19/2,560	peruvoside, convallatoxin (glycosides)	[19]
		MYH6+ nuclei count	3/280	BIO (GSK3 inhibitor) + SB203580 (p38 inhibitor) + SU1498 (FLK1 inhibitor)	[20]
	neonatal mouse CMs		43/378	TG003 (CLK/DYRK inhibitor)	[21]
	zebrafish embryos	FUCCI: cdt1/geminin	104/1,200	calcitriol, alfacalcidol (vitamin D analogs)	[22]
gene	fetal mouse CMs	EdU, pHH3	146/15,500	ENG	[23]
	human iPSC-CPCs	nuclei count	12/923	FGF16	[24]
	neonatal mouse CMs	pHH3	3/15	CDK1 + CCNB + CDK4 + CCND	[25]
microRNA	human iPSC-CMs	EdU	96/875	67 microRNAs target Hippo pathway	[26]
	neonatal rat CMs		204/875	microRNA-590, microRNA-199a	[27]

Abbreviations: *ADRA1* alpha 1 adrenergic receptor, *CCNB* cyclin B, *CCND* cyclin D, *CDK1* cyclin dependent kinase 1, *CDK4* cyclin dependent kinase 4, *CLK* CDC-like kinase, *CM* cardiomyocyte, *CPC* cardiac progenitor cells, *DYRK* dual specificity tyrosine phosphorylation regulated kinase, *EdU* 5-Ethynyl-2′-deoxyuridine, *ENG* endoglin, *ESCMs* embryonic stem cell-derived cardiomyocytes, *FGF16* fibroblast growth factor 16, *FOXO* forkhead box O, *FUCCI* fluorescent ubiquitination-based cell cycle indicator, *GSK3* glycogen synthase kinase-3, *HOXB13* homeobox B13, *iPSC* induced pluripotent stem cells, *JAK1* janus kinase 1, *LTCC* L-type calcium channel, *MCT1* monocarboxylate transporter 1, *MEIS1* meis homeobox 1, *mTORC* mechanistic target of rapamycin complex, *MYH6* myosin heavy chain 6, *p38* mitogen-activated protein kinase 14, *pHH3* phosphohistone H3, *PPARδ* peroxisome proliferator activated receptor delta, *PTEN* phosphatase and tensin homolog, *TGFBR* transforming growth factor beta receptor, *YAP* yes-associated protein

Many screens focus on specific molecular targets. For example, initial studies identified Yes-associated protein (YAP) activation as a key proliferative signal [28], leading to a subsequent small molecule screen targeting this pathway [16]. A separate screen of neonatal ECM constituent components identified agrin and showed intramyocardial injection enhances YAP activity and facilitates cardiac regeneration following MI [29]. Similarly, Meis1 and Hoxb3 were first recognized as regulators of CM proliferation [30] with later studies identifying FDA approved drugs that bind these transcription factors to modulate regenerative activity [10] through advances in structure-based virtual screening and biophysical validation [31].

Combination therapies may offer advantages over single-target drugs, as they can address the multifaceted nature of CM proliferation and regeneration [17, 20, 25]. However, a critical challenge remains in determining whether findings are scalable across species and developmental stages. For instance, a screen identified a CLK/DYRK inhibitor that promoted CM proliferation [21], yet a different DYRK1A inhibitor was effective only after MI [32], emphasizing the existence of additional factors that are not appropriately modeled in the initial screen. While translation to humans is the ultimate goal [33], a compound’s efficacy does not necessarily need to be conserved across all species and models.

Beyond phenotypic screening, multiomic approaches provide mechanistic insights into CM proliferation by integrating transcriptomic, proteomic, and epigenomic data. Mechanistic insights provided by multiomic approaches increase the likelihood of generalizing the results in subsequent experiments. For example, one study screened over 5,000 small molecules in hiPSC-CMs to identify LTCC as a target, supported by multiple FDA-approved drugs [15]. With the downstream mediators unknown, in a subsequent study they integrated multiomics to discover that VEGFR2, PI3K, and JNK mediate how multiple small molecules induce cell cycle activity [34]. Further implicating an important role for LTCCs in regulating cardiac regeneration, another drug screen reproduced the efficacy of LTCC inhibition in stimulating CM cell cycle activity not only in CMs but also cardiac organoids and with genetic inhibition in adult mouse heart and human cardiac slices [11]. A logic-based network model of CM proliferation signaling has been used for virtual knockdown screens to identify and validate that c-Myc is a downstream mediator of YAP-induced CM proliferation [35]. This model further presents an opportunity for in silico drug screening, similar to previous studies that have identified FDA approved drugs that attenuate CM hypertrophy [36].

## Challenges in Drug Discovery

### Is Targeting CM Proliferation Sufficient As a Therapy?

While stimulating CM proliferation can restore CM quantity, it may not translate to improved cardiac function. One limitation is that immature CMs may fail to integrate properly with existing myocardium or even form an arrhythmogenic substrate due to differences in electrophysiological properties and contractile function. Additionally, most adult CMs are binucleated or polyploid, which may restrict their ability to re-enter the cell cycle and might limit the regenerative capacity of the heart [37, 38]. Although advancements in organoid models have helped address some of the maturation and contractility limitations of 2D culture systems [39], heterotypic cell interactions and in vivo environmental factors remain critical considerations. CM proliferation alone does not account for the complex interactions between CMs, endothelial cells, fibroblasts, and immune cells, all of which play essential roles in cardiac repair. Paracrine signaling from non-CMs can modulate CM proliferation, as demonstrated by the influence of regulatory T cells [40], macrophages [41, 42], and fibroblasts [43]. Co-targeting other cell types may provide a more effective regenerative strategy, such as reprogramming fibroblasts into CMs [44, 45], or promoting endothelial-driven angiogenesis, which precedes heart regeneration post-MI [46]. An alternative approach involves tissue-engineered cardiac muscle, where bioengineered cardiac patches containing proliferative CMs could complement direct CM proliferation strategies [47]. Given these complexities, effective regenerative therapies may require a multifaceted approach that integrates CM proliferation with strategies to enhance cell-cell communication, vascularization, and tissue engineering to address other critical processes of regeneration such as scar resorption.

### Will CM Proliferation Translate?

A major challenge in drug screening is the generalizability of results, as many high-throughput screening hits fail to translate across different models or species. Variability in cell culture conditions, experimental protocols, and genetic backgrounds can contribute to inconsistencies, underscoring the need for more rigorous cross-validation and standardization. One approach for integrating across contexts and candidates is computational modeling, which is now widely used in drug discovery [48] and development [49]. Despite their utility, computational models have inherent limitations, as their predictive accuracy is highly dependent on the quality and completeness of input data. While in silico approaches can efficiently identify potential drug candidates, their translational relevance is constrained by gaps in biological knowledge and model assumptions. Efforts to improve prediction accuracy include integrating single-cell RNA sequencing to enhance cell type-specific [50] and temporal predictions [51], as well as leveraging multiomic approaches to capture complex regulatory networks [52]. To maximize clinical relevance, patient-derived samples are essential for validating computational predictions [33]. In particular, human iPSC-derived CMs offer a promising platform for studying CHD and its complications through patient-specific, de novo-generated models [53]. Additionally, specificity and safety concerns remain critical barriers to clinical translation. Uncontrolled CM proliferation can lead to severe complications such as arrhythmias [54], dilation due to lack of contractile force, or development of heart failure [55], emphasizing the need for precise regulation of proliferative signaling. Encouragingly, transient stimulation of CM proliferation has shown potential to mitigate these risks [51]. However, off-target effects of candidate drugs could result in adverse outcomes, including cardiac hypertrophy, conduction defects, or toxicity to non-cardiac organs, including tumors. Addressing these challenges requires a multifaceted approach that combines computational predictions with experimental validation and stringent safety assessments.

### Translational Success in Academia and Industry

Based in part on the challenges noted above, there have been both progress and barriers in translating candidate therapeutics to large animal models and the pharmaceutical industry. Noteably, the Giacca, Zhang, Sadek, and Martin labs have all demonstrated enhanced cell cycling with candidates in pig models of ischemia-reperfusion. An early success was the discovery of microRNA-199a through functional screening, validated in mouse models of MI [27]. The Giacca lab translated microRNA-199a overexpression via adeno-associated virus to a to a pig model of ischemia-reperfusion, which remarkably increased stimulated CM proliferation markers, muscle mass, and cardiac function at 12 days post-injury [54]. However, they also reported that by 28 days there was sudden arrhythmic death and myocardial infiltration of myoblast cells [54], providing a cautionary tale for the field. Follow-up studies from the same group revealed that the initial benefits of microRNA-199a expression in pigs relied on early delivery, with reduced benefits from later delivery [56]. Fan et al. took an orthogonal approach, loading GSK3β inhibitor CHIR and FGF1 onto nanoparticles, building on prior findings that this combination promotes CM proliferation in human iPSC-CMs [57]. While the CHIR-FGF1 nanoparticles improved cardiac function in both mice and pigs subjected to ischemia-reperfusion, these effects were attributed to enhanced angiogenesis and reduced apoptosis rather than CM proliferation.

More recent studies have demonstrated benefits that appear more directly tied to CM proliferation. Both with combinations of paromycin and neomycin in the Sadek lab and with modified mRNA for cyclin D2 in the Zhang lab, these perturbations enhanced markers of CM proliferation, had smaller infarct, and improved cardiac function in pig models of ischemia-reperfusion [10, 58]. Extending their earlier work in mice, the Martin lab used adeno-associated virus to knockdown the Hippo inhibitor Salvador in pigs after ischemia-reperfusion, finding increased nuclear YAP translocation, CM proliferation markers, and long-term functional benefits [59]. Motivated by concerns from other studies above, they performed more detailed analysis of arrhythmogenic events, which were fortunately decreased by Salvador knockdown [59].

Several of these advances in CM proliferation have progressed to intellectual property, industry collaboration, and clinical activity. For example, several impactful studies have corresponding granted patents on pro-proliferative targets such as neuregulin [JP6134190B2], the Hippo/dystrophin complex [60], agrin [61], mevalonate biosynthesis [62], and FGF9 [63]. While these are primarily granted to academic institutions, smaller companies such as Jaan Biotherapeutics [64] and Five Prime Therapeutics [63] have pending patent applications, and YAP Therapeutics [65] has an active development program. While larger pharmaceutical companies including AstraZeneca [12, 14, 15, 34, 39, 66, 67], Tenaya [25, 51], Novartis [68], Amgen [69], and Bristol Meyers Squibb [70], have published work related to CM proliferation, these publications do not necessarily reflect active drug development efforts. To our knowledge, the only clinical trial specifically focused on CM proliferation is based on the findings from the Kuhn lab on enhanced CM proliferation markers with beta blocker treatment in human tissue [71]. Their trial is testing whether the beta blocker propranolol will enhance CM proliferation markers and cardiac function in patients with the CHD Tetralogy of Fallot [72]. Together, the above translational activities demonstrate progression to intellectual property and clinical trials. However, it appears this is a challenging space due not only to the complexity and scale required for trials for heart disease but also the complexity of CM proliferation biology noted in the previous sections.

## Conclusion

**Fig. 1:**
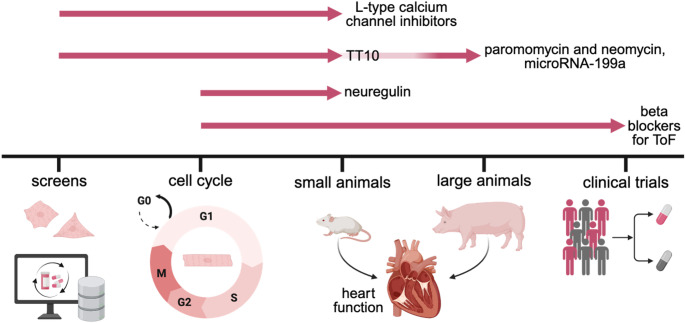
Advances in cardiac regeneration therapies. Overview of screening strategies and experimental models used to develop cardiomyocyte proliferation-based therapies across the translational pipeline. Early-stage approaches including computational modeling, in vitro phenotypic assays, and cell cycle analysis enable high-throughput screening and mechanistic discovery. These findings are then validated in small animal models [11, 16, 27, 74, 75], with subsequent testing in large animal systems [10, 54] and human preclinical assessments. Ultimately, candidate regeneration therapies are evaluated in clinical trials [76]. ToF Tetralogy of Fallot. Created in BioRender. Wintruba, K. (2025) https://BioRender.com/6z2krl7

While no current regeneration approach has fully translated to treating the diseased human heart [73], recent advances have brought the field significantly closer to developing clinically relevant therapeutics (Fig. 1). Advances in high-throughput drug screening have uncovered candidate compounds and molecular pathways with regenerative potential. However, translating these discoveries into safe and effective therapies remains a major challenge. The complexity of cardiac repair underscores the need for a deeper mechanistic understanding of heart regeneration. Continued investment in basic research is essential to elucidate the regulatory networks governing CM proliferation, refine in vitro and in vivo models, and inform the design of targeted drug discovery strategies. Ultimately, sustained efforts at the intersection of basic science and translational research will be critical to advancing regenerative therapies for heart disease.

## Key References

- Ahmed MS, Nguyen NUN, Nakada Y, et al. (2024) Identification of FDA-approved drugs that induce heart regeneration in mammals. Nat Cardiovasc Res 3:372–388.
  - This study used in silico screening to identify drugs that inhibit the transcriptional activity of Meis1 and Hoxb13, and subsequently validated enhanced cardiomyocyte proliferation and improved cardiac function in adult mice and pigs following myocardial injury.
- Devilée LAC, Salama A bakr M, Miller JM, et al. (2025) Pharmacological or genetic inhibition of LTCC promotes cardiomyocyte proliferation through inhibition of calcineurin activity. NPJ Regen Med 10:1.
  - This study used cardiac organoids to screen for compounds that inhibit L-type calcium channels and further identified that genetic overexpression of RRAD promotes cardiomyocyte proliferation in adult mice.

## Data Availability

No datasets were generated or analysed during the current study.
